# Trends in Growth and Maturation in Children with Cystic Fibrosis Throughout Nine Decades

**DOI:** 10.3389/fendo.2022.935354

**Published:** 2022-07-12

**Authors:** Kelly A. Mason, Alan D. Rogol

**Affiliations:** Division of Pediatric Endocrinology, University of Virginia, Charlottesville, VA, United States

**Keywords:** cystic fibrosis, growth, height, weight, puberty, body composition

## Abstract

Since cystic fibrosis (CF) was first described in 1938, there have been many discoveries and innovations in the field, each having a profound impact on survival, growth and quality of life. For example, the introduction of enteric-coated pancreatic enzyme microspheres increased fat absorption and improved nutritional status. Early detection of CF through newborn screening facilitated prompt nutritional intervention for infants at high risk of malnutrition. Use of anti-pseudomonal therapy, such as inhaled tobramycin, increased weight gain and pulmonary function in addition to reducing pulmonary exacerbations. Similarly, DNAse and hypertonic saline improved pulmonary function and reduced exacerbations. The identification of the *CFTR* gene and its protein product were fundamental in understanding the pathophysiology of CF and paved the way for advances in both diagnosis and management. In fact, CFTR modulator therapies have revolutionized the care for individuals with CF. Here, we examine the impact of these interventions on the nutritional status, growth and pubertal maturation of children and adolescents with CF.

## Introduction

Cystic Fibrosis (CF) affects nearly 70,000 individuals worldwide. It is caused by an autosomal recessive mutation in the cystic fibrosis transmembrane conductance regulator (*CFTR*) gene, which results in a dysfunctional CFTR protein. This, in turn, impairs chloride ion transport to the cell surface, resulting in viscous secretions in the lungs, pancreas, intestine and hepatobiliary ducts, leading to obstruction and fibrosis (1). Since its initial description by Dorothy Andersen in 1938 (2), there have been many landmark discoveries that have led to remarkable improvements in life expectancy and quality of life.

For decades, chronic infection, malabsorption of essential nutrients, inflammation and the frequent use of corticosteroids set the child with CF on a course of diminished weight gain and linear growth restriction. It should be noted that increased energy requirements (expenditure) due to inflammation and chronic pulmonary disease also contribute to the energy deficit. Growth restriction may even begin *in utero* since the birthweight of children with CF is approximately 250 g less than that of healthy newborns (3). It may be that the absent CFTR affects placental function.

Over the decades the treatment goals have been to optimize pulmonary function and growth through proper nutrition and reduced inflammation. We aim to characterize the trends in growth and maturation over time. Notably, many studies report anthropometric data differently, using weight or height-for-age or weight-for-height as a % or z-score or % of reference median. In their study of 13,116 children with CF, Lai et al. discovered inconsistencies in classifications when using various criteria, underscoring the importance of standardized definitions (4).

Nevertheless, it is evident that interventions such as multi-disciplinary care and the introduction of newborn screening have contributed to the increased growth of children with CF, demonstrated by an increase in the average height z-score at age 6 years from -0.69 to 0.39 SD (5).

## The 1930’s

Cystic Fibrosis was first described in 1938 by Dorothy Andersen who carefully reviewed the clinical histories and post-mortem examinations from 49 individuals with pancreatic fibrosis, many of whom died during infancy or early childhood (2). Infants who died within the first week of life were felt to have died from intestinal obstruction. The remaining children were characterized by failure to gain weight beginning in the neonatal period, hunger, distended abdomens, intolerance of dietary fat with large fatty stools in the absence of vomiting and diarrhea, and chronic respiratory tract infections. Andersen termed the condition cystic fibrosis of the pancreas based on the observation that the pancreatic acinar tissue “was replaced by epithelium-lined cysts containing concretions and surrounded by fibrous tissue”; there was also evidence of vitamin A deficiency. The lungs demonstrated “bronchitis, bronchiectasis, pulmonary abscesses arising in the bronchi”, and/or lobular pneumonia with S. aureus as a common “bacteriologic agent”. The oldest child in the cohort was 14.5 years (2). In 1935 Parmelee described this girl’s stature as closely approximating normal until age 11, after which time her growth was described as “retarded”, but the “development of secondary sex characteristics was not.” Her weight was reported to be considerably below average throughout much of her childhood and adolescence (6). Based on the findings in this report, this child likely had more mild disease.

## The 1940’s

Anthropometric data in children with CF throughout the 1940’s are largely unavailable.

## 1950’s

Given the short life span in those diagnosed with CF in the 1930’s and 1940’s, there are few data on growth and puberty for these children. However, the life expectancy in the 1950’s (1951-1956), increased to 59 months (7) allowing for more detailed descriptions of growth and nutritional status.

Rustin McIntosh, for example, recorded observations on a cohort of 23 patients with CF who survived to at least 10 years of age. In this group of children, malnutrition occurred more commonly during infancy compared to childhood, but height and weight were “retarded” (average height z-score 2 SD below mean and average weight 1.8 SD below mean). Height and weight were noted to increase in response to appropriate therapy for staphylococcal infections, suggesting that chronic infection and inflammation play a role in the poor growth observed in children with CF (8).

Several years later, Shwachman and Kulczycki, described a larger cohort of 105 children who were followed longitudinally for at least 5 years after diagnosis. The majority (87/105) received antibiotic therapy. Their patient population consumed a liberal diet high in protein with limited fat intake. Those who had pancreatic insufficiency took pancreatic enzyme replacement with each meal and double the typical daily dose of multivitamins. The patients were divided into groups based on the age of diagnosis and each was assigned a score based on their level of tolerated activity, physical and radiographic findings and nutritional status (Shwachman-Kulczycki or the SK score). The nutritional score was based on height and weight percentiles for age, stool characteristics, muscle mass and tone, and degree of abdominal distension. The nutritional score was not reported for all subgroups but was documented for the group diagnosed between the age of 7 and 16 years both at baseline and at follow-up. At baseline, none of the 9 children with CF were categorized as marked malnutrition, 4/9 (45%) had a weight and height for age under the 3^rd^ centile and 2 (22%) had height and weight for age over the 25^th^ centile. At follow-up 5 to 10 years later, 2 (22%) were categorized as having marked malnutrition, 4/9 (44%) had a weight and height for age less than the 3^rd^ centile and none had a height and weight for age above the 25^th^ centile, suggesting a decline in nutritional status and linear growth over time (7) (**Table 1**).

**Table 1:** Growth Data over Time.

Authors	Year	Location	Study Details	Findings
Shwachman (7)	1958	US	Longitudinal study over 5-14 yrs n=105 (Total) age 5 to >15 yrs n=9 (Number with nutritional score at baseline & 5-10 years later)	Malnourished: 0% to 22% Wt & ht <3%: 45% to 44% Wt & ht >25% 22% to 0%
Sproul (9)	1964	US	Longitudinal study over ≥ 2 yrs n=50 infancy through adolescence	Ht and wt <10% ▪ Wt affected > ht in young; stunting of ht & wt with age ↑ Wt after Abx and PERT (no impact on linear growth)
Kreiβl (10)	1972	Germany	Longitudinal study over 1-12 yrs n=53 aged 2-13.8 yrs	Ht 25-50% Wt affected > ht (M 25%; F 3-10%)
Berry (11)	1975	US	Case-control study of ‘nutritional supplement’ n=63	↑ Wt to -1SD w/early dx ↓ WV > HV after 8 yrs
Mitchell-Heggs (12)	1976	UK	Longitudinal study over 1.1-14.25 yrs n=45 aged 12-27 yrs	Ht M 25%; F 25-50% Wt affected > ht ▪ 47% wt <3%
Corey (13)	1988	US & Canada	Cross-sectional study n=1,033 aged 0-45 yrs	Pts Toronto taller than Boston (M 42% vs. 33% p<0.001; F 44% vs 33%) M Toronto heavier than Boston Attributed to diff in dietary fat & PERT
Keller (14)	2003	Switzerland	Longitudinal study for at least 11 yrs n=75 (40 born 1968-80 & 35 born 1982-96)	BMI sig ↓in young kids born earlier (p<0.047 for 2 yo; p=0.0045 for 5 yo)
Nir (15)	1996	Denmark	Cross-sectional study n=223 aged 0.75-41 yrs	Ht -0.46 SD Wt M -0.6 SD; F -0.7 SD BMI ↓ over time (98% in younger pts 83-90% in adult)
Farrell (16)	1997	US	Randomized prospective longitudinal study (Wisconsin CF Neonatal Screening Project) n=96 from age at diagnosis through 10 yrs	Length/ht & wt ↑ in those dx by NBS than non-screened ▪ Ht -0.2 vs. -1.2; p<0.001 ▪ Wt -0.5 vs -1.2; p=0.008 ▪ Persists over 10 yrs (wt p=0.04; ht p=0.02)
Lai (4)	1998	US	Cross-sectional study n=13,116	Ht 30% Wt 20%
Lai (17)	2000	US	Randomized prospective longitudinal study (Wisconsin CF Neonatal Screening Project) n=82 from age at diagnosis through 10 yrs	Ht & wt near nml in those dx by NBS (without MI) up to age 13 ▪ Ht -0.06 SD ▪ Wt -0.02 SD
Farrell (18)	2001	US	Randomized prospective longitudinal study (Wisconsin CF Neonatal Screening Project) n=104 from age at diagnosis through 13 yrs	Risk for wt or ht <10% ↓ those dx by NBS than non-screened (OR 4.12 wt & 4.62 ht)
Kastner-Cole (19)	2005	UK	Cross-sectional study n=2,987 aged 0.8 to 55.7 yrs	BMI M -0.28 to 0.8 SD; F -0.28 to 0 SD ▪ 10.2% OW or OB ▪ BMI ↓ late childhood/adolescence vs. infancy/early childhood
Hanna (20)	2015	US	Cross-sectional study n=226 aged 2-18 yrs	Malnourished (BMI <10%): 7% At risk (BMI 10-25%): 12% Healthy (BMI 25-85%): 57% OW or OB (BMI >85%): 23%
Zhang (5)	2016	US	Randomized prospective longitudinal study (Wisconsin CF Neonatal Screening Project) n=107	Adult ht sig ↑ in those dx by NBS vs. non-screened Remained sig after genetic potential adjustment (32 vs 15%; p=0.006)
Wainwright (21)	2015	Multinational	Two 24-week, randomized, double-blind, placebo-controlled trials of Lum/iva (TRAFFIC and TRASNPORT) n=1,108 ≥12 yo homozygous F508	Sig BMI ↑ from baseline in tx groups (pooled data) (tx diff 0.24-0.28 kg/m^2^ p<0.001) ▪ Not stratified by age
Stalvey (22)	2017	US	ENVISION (48 wk randomized, placebo-controlled, double-blind trial of ivacaftor) GOAL (24 wk longitudinal obs study of ivacaftor) n=83 aged 6-11 yrs w/≥1 G551D	Ht sig ↑ from baseline ▪ GOAL (z-score ↑ 0.1 SD p<0.05) ▪ ENVISION (z-score ↑ 0.17 SD from baseline p<0.001; tx diff vs placebo 0.58 SD p<0.05) Wt sig ↑ from baseline ▪ GOAL (z-score ↑ 0.26 SD p<0.0001) ▪ ENVISION (z-score ↑ 0.35 SD from baseline p<0.001; tx diff vs. placebo 0.8 SD p<0.001) HV sig ↑ ▪ GOAL (↑ of 2.1 cm/yr from pre-baseline to baseline to 3-6 mo p<0.01) ▪ ENVISION (tx diff vs. placebo 1.08 cm/yr p<0.05) WV sig ↑ ▪ GOAL (↑ of 4.54 kg/yr from pre-baseline to baseline to 6 mo p<0.0001) ▪ ENVISION (tx diff vs. placebo 3.11 kg/yr p<0.001)
Taylor-Cousar (23)	2017	Multinational	24 wk randomized, double-blind, placebo-controlled, parallel group trial of Tez/iva (EVOLVE) n= 504 ≥12 yrs homozygous F508	No sig diff in BMI ↑ between tx & placebo
Ratjen (24)	2017	Multinational	24 wk randomized, double-blind, placebo-controlled trial of Lum/iva n=204 6-11 yrs homozygous F508	No sig diff in BMI ↑ between tx & placebo groups
Middleton (25)	2019	Multinational	24 wk randomized, double-blind, placebo-controlled trial of Elex/tez/iva n= 403 ≥12 yrs single F508 & minimal function genotype	Sig BMI ↑ vs. placebo (tx diff 1.04 kg/m^2^ p<0.001) Not stratified by age
Heijerman (26)	2019	Multinational	4 wk randomized, double-blind, active-controlled trial of Elex/tez/iva n=107 ≥12 yrs homozygous F508	Sig ↑ wt (tx diff 1.6 kg p<0.001) Sig ↑BMI (tx diff 0.6 kg/m^2^ p<0.0001) Not stratified by age
Owen (27)	2021	UK	Cross-sectional study n=37 aged 5-8 yrs	Ht, BMI, FEV_1_ within nml Wt and body comp sig ↓ reference data ▪ 5% FFMI z-score <1 despite optimal BMI
CFF Annual Report 2020 (28)	2020	US	n=31,411 (13,444 children)	<2 yo ▪ Wt 0-2 years: 44.5% ▪ Ht 0-2: 31.5% ▪ WFL 0-2 years: 62.8% 2-19 yo ▪ Wt 2-19 years: 51.9% ▪ Ht 2-19: 38.7% ▪ BMI 2-19 years: 61.4%
Marks (29)	2021	US	Retrospective case-control study n=3,655	Max ht between 2-4 yrs highly correlated w/max adult height (r=0.64)

Wt, weight; ht height; abx, antibiotic; PERT, pancreatic enzyme replacement therapy; dx, diagnosis; WV, weight velocity; HV, height velocity; M, male; F, female; pts, patients; diff, difference(s); sig, significantly; yo, year-olds; NBS, newborn screen; yrs, year(s); nml, normal; OW, overweight; OB, obese; tx, treatment; mo, month(s); lum/iva, lumacaftor/ivacaftor; wk, week(s); obs, observational; tez/iva, tezacaftor/ivacaftor; elex/tez/iva, elexacaftor/tezacaftor/ivacaftor; comp, composition; WFL, weight-for-length; max, maximum.

The birthweights of infants with CF were significantly below those of both the general population (30) and unaffected siblings (31), suggesting that the slow growth was not solely due to infections, pancreatic insufficiency or nutritional status, but began *in utero.*

## The 1960’s

The 1960’s marked the beginning of a specific focus on growth in children with CF. Sproul and Huang studied the growth patterns in 50 children and noted a period of accelerated weight gain after initiation of therapy that included antibiotics and pancreatic enzyme replacement therapy (PERT), with the longest duration of effect occurring when therapy was initiated during infancy. They did not find an effect on linear growth despite a minimum observation period of 2 years. The authors observed that the median height and weight for all age groups were under the 10^th^ centile with weight more affected than height in the pre-school and school-age children and growth “retardation” more prominent in the preadolescent and adolescent age groups, a finding that was attributed to lack of the pubertal growth spurt (**Table 1**). Skeletal maturation was evaluated in 40 of the 50 children and was “retarded” in 25%. Notably, the authors also observed an inverse relationship between severity of respiratory disease and growth (p=0.005 for height; p<0.001 for weight), though the methods used for categorizing respiratory status were not reported (9) (**Table 2**).

**Table 2:** Relationship between Growth and Pulmonary Function.

Author	Year	Location	Study Details	Findings
Sproul (9)	1964	US	Longitudinal study over ≥ 2 yrs n=50 infancy through adolescence	Severity of respiratory disease assoc w/ht (p=0.005) & wt (p<0.001)
Kreiβl (10)	1972	Germany	Longitudinal study over 1-12 yrs n=53 aged 2-13.8 yrs	Wt assoc with SK score (r=0.53) ▪ No assoc between SK score & ht
Berry (11)	1975	US	Case-control study of ‘nutritional supplement’ n=63	Wt & ht pos correlated to clinical score ▪ Stronger assoc with wt than ht
Greco (32)	1993	Italy	Longitudinal study 3-14 yrs n=28 aged 4.5 to 17.5 yrs	Resp and GI events coincide with descending phase of HV and WV
Byard (33)	1994	US	Longitudinal study n=230 aged 4.75 to 22.25 yrs	RV/TLC assoc with HV at TO, PHV and PHV +2 yr
Nir (15)	1996	Denmark	Cross-sectional study n=223 aged 0.75-41 yrs	Sig assoc between BMI & FEV_1_ (p<0.0001)
Konstan (34)	2003	US	Longitudinal study through at least age 6 yrs n=931	FEV_1_ highest in those who maintained wt >10% from 3-6 yrs FEV_1_ lowest in those whose wt remained <10% from 3-6 yrs
Stallings (35)	2008	US	NA	FEV_1_ (~≥80%) assoc with BMI ≥50%
Sheikh (36)	2014	US	Cross-sectional study n=208 aged 5-21 yrs	LBMI-z & BMI-z pos assoc with FEV_1_ (not FMI-z) LBMI-z more strongly assoc with FEV_1_ vs. BMI-z ▪ F p<0.0001 vs. p=0.001 ▪ M p<0.0001 for both
Hanna (20)	2015	US	Cross-sectional study n=226 aged 2-18 yrs	FEV_1_ lowest in nutritional failure p<0.0005 ▪ No sig diff in other 4 wt groups including normal wt vs. obese
Owen (27)	2021	UK	Cross-sectional study n=37 aged 5-8 yrs	WB FFMI & BMI pos assoc with FEV_1_ WB FFMI more strongly assoc with FEV_1_ vs. BMI (p=0.02 vs. p=0.08)
Sanders (37)	2021	US	Longitudinal study through age 7 yrs n=5,388	FEV_1_ higher in children at 6-7 yo who maintained ht >50% vs. those with ht <50% after adjusting for BMI

Assoc, associated; ht, height; wt, weight; SK, Shwachman- Kulczycki score; resp, respiratory; GI, gastrointestinal; HV, height velocity; WV, weight velocity; RV, residual volume; TLC, total lung capacity; TO, take-off; PHV, peak height velocity; yr, year(s); LBMI-z, lean body mass index z-score; pos, positively; FMI-z, fat mass index z-score; F, female; M male; sig significant; diff difference; WB FFMI whole body fat free mass index; yo years old.

## The 1970’s

By 1974, life expectancy reached 16 years (38) and several investigators began to describe the growth patterns and pubertal maturation of children and adolescents with CF (10–12, 38). Antibiotic agents and treatment intensity improved between the 1960’s and 1970’s (10), such that standard therapy in the 1970’s included use of antimicrobials based on susceptibility and clinical status, aerosol inhalations or nightly mist therapy, postural drainage and chest physiotherapy, vitamin supplementation and PERT (11, 38). In 1979, encapsulated enteric-coated pancreatic enzyme microspheres were introduced. This formulation was designed to reduce gastric acid and pepsin-mediated inactivation, thereby delivering more active enzyme to the duodenum (39) and was shown to be effective in the treatment of pancreatic insufficiency (40).

In 1972, Kreiβl and colleagues evaluated growth parameters in a cohort of 53 patients. Similar to Sproul and Huang, their group identified an association between weight percentiles and disease severity as determined by Shwachman-Kulczycki scores. However, they found no association between disease severity and height (**Table 2**). The median heights in this particular group ranged from the 25^th^ to the 50^th^ centile, with a normal height velocity in all but 7 of the 53 children. As observed in previous studies, weight tended to be more affected than height (**Table 1**). Weight velocity, however, was considered normal in all but 11 of the 53 children (10).

As in the study by Kreiβl, Mitchell-Heggs and colleagues reported a median height at the 25^th^ centile in boys and between the 25-50^th^ centiles in girls. The authors noted that the mean weight percentiles tended to be lower than the mean height percentiles for most (**Table 1**). They also reported that puberty tended to be delayed, but there was no significant relationship between skeletal maturity and disease severity (12).

Results from Berry et al. were similar to those that Sproul and Huang reported in 1964 (9). They too observed an increase in weight following early diagnosis that stabilized 1 SD below the mean from 2 to 8 years of age. This was followed by a gradual decline in height and weight velocity with weight more significantly affected than height (11) (**Table 1**).

In 1976, Stern et al. reported on a cohort of 95 patients and noted that weight-for-height was generally ‘deficient’ with weight often under the 3^rd^ centile and height below average. Menarche was noticeably delayed with a mean age of >14 years (38).

Throughout the 1970’s, many studies revealed that weight was more significantly affected than height and that puberty and/or skeletal maturity tended to be delayed.

## The 1980’s

By the early 1980’s the mean survival for those with CF increased to 19 years in the United States and 21 years in Canada (13). One notable change to the care of individuals with CF was early detection through newborn screening. In 1985, a comprehensive evaluation of a newborn screening program in Wisconsin began (16). There were also significant changes to nutrition. In the early 1980’s, for example, enteric-coated pancreatic enzyme microspheres significantly increased fat absorption over conventional pancreatic enzyme formulations (41). A seminal paper by Corey and colleagues in 1982 demonstrated a significant growth discrepancy between children treated at the CF center in Toronto and those treated in Boston and speculated that these discrepancies were due to differences in PERT dosing and dietary fat intake. Both males and females in Toronto were significantly taller than those in Boston; males in Toronto were also significantly heavier than those treated in Boston, where dietary fat intake was limited (13) (**Table 1**). These findings led to the adoption of the high-calorie, high-fat diets that became fundamental in the care of children with CF.

Soutter et al. reported that most of the children in their cohort had a weight under the 50^th^ centile with a decline in late childhood and pre-adolescence. However, weight and height velocity nearly doubled in those receiving overnight gastrostomy tube feeds, highlighting the importance of caloric (energy) intake. The mean height in their cohort was under the 50^th^ centile (often ranging between the 25 and 50^th^ centiles) (42). These data are similar to those published by Kreiβl and Mitchell-Heggs in the 1970’s but reflect a significant improvement from those reported by Sproul in the 1960’s (9, 10, 12).

Similar to Soutter et al., Keller and colleagues noted that the mean height in their population was significantly lower than the general population, as were weight and BMI z-scores. However, BMI z-scores in young children were significantly higher in the cohort born in the 1980’s and 1990’s compared to the cohort born in the 1960’s and 1970’s, supporting an overall improvement in nutritional status over time (14) (**Table 1**).

Puberty in individuals with CF tended to be delayed in the 1980’s with reports of delayed peak height velocity (PHV) (15 years) in boys (43) and delayed menarche (14.5 years) in girls (44). Some authors attributed the delayed puberty to disease severity and nutritional status (44), while others found no such association (45).

Throughout the 1980’s, height, weight and BMI in children with CF remained below the general population, though they appeared to be greater than earlier cohorts. The median survival had also increased such that it approximated 30 years by the late 1980’s (15).

## The 1990’s

In the 1990’s there was a focus on airway clearance through use of inhaled DNAse to thin the DNA-rich viscous secretions as well as anti-pseudomonal therapy. In a landmark study in 1994, inhaled DNAse (Pulmozyme) improved pulmonary function more than placebo and resulted in fewer pulmonary exacerbations (46). Use of inhaled tobramycin, an anti-pseudomonal therapy, every other month improved pulmonary function, reduced the need for IV antibiotics, and increased weight compared to placebo (47).

The 1990’s appear to be a turning point with a general improvement in growth patterns. In fact, the mean height of Swedish children approximated that of the general population (z-score of -0.3 SD) by age 5 y (48). Early identification of CF through newborn screening likely contributed to the improvement in growth observed throughout the decade. In fact, infants diagnosed by newborn screening (mean age 12 weeks) had significantly greater length or height z-scores (-0.2 SD vs. -1.2 SD), weight z-scores (-0.5 SD vs. -1.2 SD) and head circumferences than those identified by symptoms (mean age 72 weeks) (16). Furthermore, those diagnosed by newborn screening were taller and heavier up to 10 years later compared to those diagnosed by symptoms alone (16) (**Table 1**).

Authors from the UK reported mean weight z-scores ranging from -0.25 and -0.5 SD in their population under age 23 (49). Although these were lower than the general population, this reflects a general improvement from data published in the 1970’s (11, 38). However, as seen in previous studies (11, 42), these authors also reported a decline in weight z-scores after the first decade of life with BMI z-scores declining from 0 to -0.5 SD to -0.5 to -1 SD (49).

Nir et al. studied a cohort of 223 patients in Denmark after high fat diets and routine anti-pseudomonal therapy became standard. Their group also observed a decline in BMI over time, such that BMI values were lower than the general population by late childhood and early adolescence (**Table 1**). In addition, they identified a strong positive correlation between nutritional status (as indicated by BMI) and FEV_1_ (p<0.0001) (15) (**Table 2**). Greco et al. reported a regular intermittent (pulsatile) pattern in height and weight velocity with most respiratory or GI events coinciding with a descending phase of height and weight velocity (32) (**Table 2**). Similarly, Byard demonstrated a significant correlation between height velocity and pulmonary function (residual volume/total lung capacity) at the onset of the pubertal growth spurt, PHV and 2 years later (33) (**Table 2**).

Lai et al. analyzed data from 13,116 children participating in the National Cystic Fibrosis Patient Registry in 1993 and reported that the median height fell along the 30^th^ centile with weight more significantly affected (20th centile), as observed in previous studies (4, 10–12, 38) (**Table 1**). Malnutrition, as defined by height or weight under the 5^th^ centile was common during infancy. Similar to Nir et al., Lai and colleagues found that malnutrition was also common in adolescence (15). More girls (29%) than boys (19%) were short (height less than the 5^th^ centile) between the ages of 11-14 with the opposite trend noted between the ages of 15-18 (4), potentially related to differences in pubertal timing.

Indeed, Byard reported on 230 children in the US and noted that PHV was lower in magnitude and occurred later than the general population (33). Similarly, in a small study of 17 girls with CF, both age at PHV and age of menarche were significantly later than the general population (50).

Previous studies from the 1960’s and 1970’s demonstrated an increase in weight upon initiation of appropriate therapy for CF (9, 11), likely due, in part, to the association between growth and disease severity identified by several groups in the 1990’s (15, 32, 33).

Despite the overall increase in growth parameters in the 1990’s, additional studies continued to report a general decline in weight z-scores after the first decade of life (4, 15, 49), comparable to findings in earlier decades (11, 42).

## The 2000’s

Airway clearance and anti-pseudomonal therapy remained important therapeutic factors in the 2000’s. Similar to inhaled DNAse therapy, inhaled hypertonic saline, used to rehydrate the airway surface and improve mucociliary clearance, reduced pulmonary exacerbations and improved pulmonary function over placebo (51). In addition, oral azithromycin was associated with a reduced risk of pulmonary exacerbations and improved pulmonary function over placebo for those with chronic pseudomonas aeruginosa infections (52).

Studies on newborn screening continued to show a benefit of early detection. The mean height (-0.6 SD) and weight z-scores (-0.23 SD) of those without MI (less likely to be identified early by symptoms alone) approximated those of the general population (17) (**Table 1**). Furthermore, when studied for up to 13 years, the odds ratios for the risk of height or weight under the 10^th^ centile in the non-screened group compared to the screened group were 4.62 and 4.12 respectively (18, 53) (**Table 1**). Data continued to support an association between growth and pulmonary function (34) and, in 2008, evidence-based practice recommendations advocated for individuals aged 2-20 years to maintain a BMI ≥50%, given the association with improved pulmonary function (35) (**Table 2**).

Pulmonary function was also associated with PHV. Specifically, those classified as having severe disease based on a diminished FEV_1_ had a PHV that was delayed and of lower magnitude than those with milder disease (54).

In a study of 84 Polish children, mean height SD was -0.57 with only 11% of the population having a height z-score more than 2 SD below the mean (55), a stark contrast from the nearly 45% in a notably smaller cohort reported by Shwachman and Kulczycki almost 5 decades earlier (7). The mean BMI z-score in this cohort was -0.77 SD with 33% being classified as malnourished (BMI <10%) and 1 child categorized as obese (55). In the UK, 10.2% of children were categorized as overweight or obese with mean BMI z-score ranging from -0.28 to 0.8 SD in boys and -0.28 to 0.00 SD in girls (19). As observed in earlier studies (15, 49), mean BMI tended to be lower in later childhood and adolescence compared to infancy and early childhood (19) (**Table 1**).

Data published in 2004 demonstrated that height and weight z-scores were not significantly different in a cohort of 15 non-oxygen dependent children with CF compared with their age and sex-matched controls. Furthermore, there were no significant differences in body fat percentage, fat mass, fat-free mass or resting energy expenditure between the two groups (56).

Studies from the 2000’s continue to demonstrate an association between growth and pulmonary function. In general, height and weight appear greater than those recorded in previous decades (**Table 2**) with some children meeting criteria for overweight or obesity (19, 55).

## The 2010’s

Nationwide newborn screening in the United States occurred by 2010 (57). This allowed for early detection and early nutritional intervention for infants with CF. It also permitted an opportunity for aggressive PERT dosing, which was associated with a favorable change in weight-for-age and weight-for-length at age 2 years (58).

In a multi-center longitudinal observational cohort study of 231 infants diagnosed with CF by newborn screening (BONUS study), infants achieved a weight z-score of -0.04 SD by 12 months of age, a significant increase over a cohort who did not undergo screening 20 years earlier (59). Although length z-scores increased over the earlier cohort, they remained low through 12 months of age (**Table 1**). In an additional study, adult height was significantly greater in those diagnosed by newborn screening (50 vs. 29^th^ centile) compared to those diagnosed by symptoms, a finding that remained significant after adjusting for genetic height potential (32 vs. 15^th^ centile) (5) (**Table 1**).

Similarly, VanDevanter reported an increase in mean height z-scores and pulmonary function that occurred in parallel to an increase in the proportion of infants diagnosed by newborn screening between 1994 and 2012 (60).

As with studies in the 2000’s (19, 55), Marília da Silva Garrote and colleagues in Brazil categorized nearly 1/3 of their population as malnourished (BMI <10%) and approximately 15% as overweight or obese (defined as BMI >75%). The risk of colonization with pseudomonas aeruginosa was 2.3 times higher in those who were malnourished compared to those who were not (61), a finding that may have contributed to the overall improvement in nutritional metrics and possibly inflammatory status with the initiation of anti-pseudomonal therapy.

These data are in contrast to those presented by Hanna and Weiner, in which only 7% of children in a single United States center were categorized as malnourished (BMI <10%), over half as healthy (BMI 25-85%) and nearly a quarter as overweight or obese (BMI >85%) (20) (**Table 1**).

By the early 2010’s, the median childhood BMI reported in the Cystic Fibrosis Foundation Annual Report approached the 50% (28). This decade marked the beginning of more targeted therapies, namely CFTR-modulatory agents (**Table 3**). The first CFTR potentiator, ivacaftor, was FDA approved in 2012 for use in individuals ≥6 years of age with relatively uncommon (gating) mutations, comprising approximately 5% of the CF population (62, 63).

**Table 3:** CFTR Mutations and Modulator Therapies.

Mutation Class (more severe to less severe)	Impact on CFTR	CFTR Modulator Therapy	Example of CFTR Modulator
I	Defect in CFTR protein production		
II (example Phe508del)	Ineffective CFTR protein processing → ineffective trafficking to cell surface	Corrector +/- potentiator	Ivacaftor/lumacaftor Tezacaftor/ivacaftor Elexacaftor/tezacaftor/ivacaftor
III (example G551D)	Inability of CFTR protein to remain open “gating mutations”	Potentiator	Ivacaftor
IV	Ineffective CFTR protein with reduced chloride transport	Potentiator	Ivacaftor
V	Decreased CFTR synthesis		
VI	Reduced CFTR stability		

Several studies demonstrated a significant increase in growth and nutritional status in children with at least one G551D mutation treated with ivacaftor. Specifically, ivacaftor treatment for 48 weeks resulted in more weight gain (1.9 kg, p<0.001) and a greater increase in BMI z-score (0.45 SD, p<0.001) in children aged 6-11 years when compared to placebo (ENVISION) (64).

Stalvey and colleagues performed a *post-hoc* analysis of 83 children ages 6-11 years enrolled in the ENVISION and the GOAL study, a 6-month longitudinal observational study of ivacaftor therapy in those with at least one G551D mutation. Height and weight z-scores increased significantly from baseline in both studies (height z-score increase of 0.1 SD in GOAL and 0.17 SD in ENVISION; weight z-score increase of 0.26 SD in GOAL and 0.35 SD in ENVISION). Height velocity increased significantly in both studies (increase of 2.1 cm/yr between 3-6 months of treatment in GOAL and 1.08 cm/yr greater than placebo in ENVISION). Furthermore, weight velocity was significantly greater in the ivacaftor group compared to the placebo group in ENVISION (treatment difference of 3.06 kg/yr in boys and 2.81 kg/yr in girls) (22) (**Table 1**).

In a 3-month observational study of 23 patients with at least one gating mutation ranging in age from 5 to 61 years, those treated with ivacaftor had a significant increase in weight, which was positively correlated to the change in FEV_1_ (p=0.028). Furthermore, there was an increase in fat free mass with a reduction in resting energy expenditure in response to ivacaftor therapy (65).

Ivacaftor therapy was also associated with a significantly greater increase in BMI and BMI z-score in children ≥ 6 years of age with non-G551D gating mutations when compared to placebo (treatment effect 0.7 kg/m^2^ and 0.28 SD respectively) (66). However, when used to treat individuals ≥ 12 years of age who were homozygous for Phe508del, there were no significant differences in change in weight or BMI between those treated with ivacaftor or placebo (67).

A few years after the FDA approval of ivacaftor, lumacafator/ivacaftor (corrector/potentiator) received FDA approval for individuals ≥12 years of age who were homozygous for the most common mutation in *CFTR*, the Phe508del (**Table 1**) (63). Data pooled from two large 24-week randomized double-blind placebo-controlled trials of lumacaftor/ivacaftor in individuals ≥12 years of age homozygous for Phe508del (TRAFFIC and TRANSPORT) demonstrated a significant increase in BMI in the treatment groups (treatment difference of 0.24-0.28 kg/m^2^). Unfortunately, these data were not stratified by age, and the specific impact in children was not reported (21) (**Table 1**). When these data were stratified by severity of pulmonary disease, the increase in BMI in the treatment group remained significant across all levels of pulmonary function (68).

In a separate 24-week randomized double-blind, placebo-controlled trial of lumacaftor/ivacaftor in children aged 6-11 years who were homozygous for Phe508del, there was an increase in BMI in both the treatment and placebo groups without a significant treatment difference (24).

Tezacaftor/ivacaftor (corrector/potentiator) was approved in 2018 for individuals homozygous for the Phe508del mutation as well as those with a single Phe508del mutation and one of 26 other mutations (62). In a study of individuals ≥12 years of age homozygous for Phe508del, BMI increased from baseline to 24 weeks in both the tezacaftor/ivacaftor and placebo groups with no significant treatment effect reported (23).

In 2019, elexacaftor/tezacaftor/ivacaftor (next generation corrector/corrector/potentiator) received FDA approval to treat the nearly 90% of individuals harboring at least 1 copy of the Phe508del mutation (62).

In a large multi-center, randomized, double-blind, active controlled trial of elexacaftor/tezacaftor/ivacaftor for individuals aged ≥12 years homozygous for Phe508del, there was a significant increase in mean weight (1.6 kg) and BMI (0.6 kg/m2) compared to those treated with tezacaftor/ivacaftor alone. Unfortunately, these data were not stratified by age and the impact on the pediatric population was not reported (26). The treatment difference in BMI (mean treatment difference of 1.04 kg/m^2^) was also significant when elexacaftor/tezacaftor/ivacaftor was compared to placebo in individuals aged ≥12 years with a single Phe508del mutation and a minimal function genotype. Again, these data were not stratified by age and the specific impact on the pediatric population could not be assessed (25) (**Table 1**). Additional study is needed to evaluate the impact of modulator therapies, including triple combination therapy, on long-term growth outcomes in children.

## The 2020’s

The nutritional status of children with CF in the 2020’s remains comparable to the general population. In fact, median weight and BMI for children and adolescents aged 2-19 are 51.9% and 61.4% respectively. Likewise, the median weight and weight-for-length during infancy and early childhood are 44.5% and 62.8% respectively (28) (**Table 1**).

Despite overall improvements in nutritional metrics, however, length and height remain below those of the general population (median length 31.5% for ages <24 months; median height 38.7% for ages 2-19 years) (28) (**Table 1**). This continues to be an important area of investigation, as early childhood height is associated with adult height. In fact, Marks et al., found that the maximum height measurements between ages 2 and 4 years are highly correlated with maximum adult height at age 18 or 19 (r=0.64) (29) (**Table 1**). Furthermore, children who maintain a height-for-age over the 50^th^ centile have a higher FEV_1_ at age 6 or 7 than those who have a height-for-age less than the 50^th^ centile for at least 1 year. The relationship between height and pulmonary function in this study was not affected by BMI adjustment (37), suggesting that linear growth alone plays an important role in pulmonary function (**Table 2**).

Recent evidence suggests that BMI may not be a reliable indicator of nutritional status in children with CF. Owen et al. reported on body composition and its association with pulmonary function in 37 pre-pubertal children (27). They found that 5% of their population had a fat-free mass index (FFMI) z-score of < -1 [near the value of hidden depletion previously published in adult CF studies (69)] despite optimal BMI z-scores. FEV_1_ was more strongly associated with whole body FFMI than with BMI (27), supporting the importance of body composition on pulmonary function previously observed in both children and adults (36, 70) (**Table 2**).

## Conclusion

Since cystic fibrosis was first described in 1938, there have been many breakthroughs in diagnosis and management. Such developments include the use of antibiotics to treat pulmonary infections, dietary modifications to increase fat and caloric intake, use of pancreatic enzymes, anti-pseudomonal therapies, early identification and intervention through newborn screening, use of mucolytics, and treatment with CFTR modulators, all of which have played a critical role in improving the life expectancy and quality of life for individuals with CF.

Growth in children with CF has historically been poor with weights and heights far below the general population, attributed to malabsorption, reduced caloric intake, increased resting energy expenditure, glucocorticoid exposure and systemic inflammation (71). In addition, historical data support an indirect impact of *CFTR* genotype on nutritional status through the association with pancreatic exocrine function (72). Whether there is a direct effect of CFTR on growth remains unknown.

Previous studies reported a greater impact on weight than height, resulting in BMI z-scores less than 0 SD. Over the last several decades, however, the weight and height z-scores seem to be increasing with some children now meeting criteria for overweight or obesity. While this could reflect an improvement in both weight and height with a more significant improvement in weight relative to height, it appears to be the result of improved weight with minimal increase in height (28, 73). One plausible explanation is a direct effect of CFTR on the GH-IGF-1 axis. However, the exact mechanism remains unclear and additional study is warranted.

While historical data demonstrate a clear association between nutritional status and lung function (34, 74, 75), more recent data support an association between height in early childhood and subsequent pulmonary function regardless of nutritional status (BMI) (37). Therefore, it will be important to identify factors that contribute to early life growth impairment and monitor the impact of highly effective CFTR modulator therapy on linear growth.

Given the minimal increase in height z-scores relative to weight, updated nutritional guidelines will be of great importance in the era of early detection and early treatment with highly effective modulator therapy to optimize growth and pulmonary function while avoiding obesity and its comorbidities. Body composition may provide better insight into nutritional status and its impact on clinical outcomes than BMI alone.

## Author Contributions

KM and AR conceived the idea for the topic, both contributed to the outline; KM wrote the first draft and KM and AR contributed to the multiple revisions. All authors contributedto the article and approved the submitted version.

## Funding

This work was supported by the Cystic Fibrosis Foundation Envision CF: Emerging Leaders in CF Endocrinology II Program [grant number MASON19GE0].

## Conflict of Interest

The authors declare that the research was conducted in the absence of any commercial or financial relationships that could be construed as a potential conflict of interest.

## Publisher’s Note

All claims expressed in this article are solely those of the authors and do not necessarily represent those of their affiliated organizations, or those of the publisher, the editors and the reviewers. Any product that may be evaluated in this article, or claim that may be made by its manufacturer, is not guaranteed or endorsed by the publisher.
